# E2F1-Mediated Upregulation of p19INK4d Determines Its Periodic Expression during Cell Cycle and Regulates Cellular Proliferation

**DOI:** 10.1371/journal.pone.0021938

**Published:** 2011-07-13

**Authors:** Abel L. Carcagno, Mariela C. Marazita, María F. Ogara, Julieta M. Ceruti, Silvina V. Sonzogni, María E. Scassa, Luciana E. Giono, Eduardo T. Cánepa

**Affiliations:** Laboratorio de Biología Molecular, Departamento de Química Biológica, Facultad de Ciencias Exactas y Naturales, Universidad de Buenos Aires, Ciudad de Buenos Aires, Argentina; Institut de Génomique Fonctionnelle de Lyon, France

## Abstract

**Background:**

A central aspect of development and disease is the control of cell proliferation through regulation of the mitotic cycle. Cell cycle progression and directionality requires an appropriate balance of positive and negative regulators whose expression must fluctuate in a coordinated manner. p19INK4d, a member of the INK4 family of CDK inhibitors, has a unique feature that distinguishes it from the remaining INK4 and makes it a likely candidate for contributing to the directionality of the cell cycle. p19INK4d mRNA and protein levels accumulate periodically during the cell cycle under normal conditions, a feature reminiscent of cyclins.

**Methodology/Principal Findings:**

In this paper, we demonstrate that p19INK4d is transcriptionally regulated by E2F1 through two response elements present in the p19INK4d promoter. Ablation of this regulation reduced p19 levels and restricted its expression during the cell cycle, reflecting the contribution of a transcriptional effect of E2F1 on p19 periodicity. The induction of p19INK4d is delayed during the cell cycle compared to that of cyclin E, temporally separating the induction of these proliferative and antiproliferative target genes. Specific inhibition of the E2F1-p19INK4d pathway using triplex-forming oligonucleotides that block E2F1 binding on p19 promoter, stimulated cell proliferation and increased the fraction of cells in S phase.

**Conclusions/Significance:**

The results described here support a model of normal cell cycle progression in which, following phosphorylation of pRb, free E2F induces cyclin E, among other target genes. Once cyclinE/CDK2 takes over as the cell cycle driving kinase activity, the induction of p19 mediated by E2F1 leads to inhibition of the CDK4,6-containing complexes, bringing the G1 phase to an end. This regulatory mechanism constitutes a new negative feedback loop that terminates the G1 phase proliferative signal, contributing to the proper coordination of the cell cycle and provides an additional mechanism to limit E2F activity.

## Introduction

A key step in cell cycle regulation is the control of the G1/S transition. This event is tightly coupled to the transcriptional control of genes involved in growth and DNA replication, which, in mammalian cells, is primarily performed by the E2F family of transcription factors [1], [2], [3], [4]. The E2F proteins, E2F1-E2F8, form heterodimers with a member of the DP protein family, DP1 or DP2. The transcriptional activity of the resulting complex is largely conferred by the E2F protein, with some members stimulating transcription (E2F1, E2F2 and E2F3a) while others inhibit it (E2F3b, E2F4, and E2F5). Some E2F family members can bind the retinoblastoma (Rb) tumor suppressor protein family, pRb, p130, and p107, and become transcriptionally inactivated by this interaction [5], [6]. Mitogenic signals promote the sequential assembly and activation of cyclin D/CDK4,6 and cyclin E/CDK2 in early and late G1, respectively, resulting in the hyperphosphorylation of pRb and release of the E2F transcription factors. In the case of E2F1, this event initiates the transcription of genes required for the G1/S transition, such as cyclin E, cyclin A, c-myc and DNA polymerase [7], [8].

Interestingly, in contrast to these growth promoting functions, E2F1 also has well-documented antiproliferative activities. E2F1 induces pro-apoptotic genes, such as caspase 3, 7, 9 and Apaf1 [9], [10], [11], [12]. Furthermore, E2F1 directly induces the expression of p14/p19ARF, resulting in p53 release from Mdm2 and its subsequent activation [13], [14], [15]. Therefore, the proliferative function of E2F1 appears to be counterbalanced by multiple self-imposed safeguard mechanisms.

Cyclin/CDK complexes are negatively regulated by small polypeptides, the CDK inhibitors (CKIs) that, in mammalian cells, fall into one of two distinct families. The INK4 family, p16INK4a, p15INK4b, p18INK4c, and p19INK4d, specifically bind to and inhibit CDK4 and CDK6 containing complexes. The Cip/Kip family, p21Cip1, p27Kip1, and p57Kip2, act as negative regulators of cyclin E/ and A/CDK2 and cyclin B/CDK1. They also act as positive regulators of cyclin D/CDK4,6 complexes by mediating their assembly early in G1 [16], [17], [18].

The four INK4 proteins share a similar structure and are equally potent as CDK inhibitors. They are however differentially expressed during mouse development, suggesting that they might have cell lineage-specific or tissue-specific functions [19]. p18INK4c and p19INK4d (p18 and p19 for the remainder of the manuscript) are expressed during embryonic development with different tissue-specificity and remain expressed at high levels in many adult tissues. In contrast, p16INK4a and p15INK4b only become detectable postnatally, and their expression increases with age [20], [21], [22]. Recent evidences support that, in addition to their role as CDK inhibitors, the individual INK4 family members would perform diverse and distinct cellular tasks. The identification of the transcription factors that regulate the expression of the INK4 genes will help understanding the physiological function(s) of the individual INK4 proteins [23]. These regulatory mechanisms, however, remain currently largely unexplored [24], [25].

The ultimate goal of the mitotic cell cycle is to guarantee that the two daughter cells inherit a complete and faithful copy of the genome of the original cell. Two types of regulatory mechanisms are critical for proper cell cycle progression. Cell cycle checkpoints ensure that cells do not progress into the next phase until the previous step is fully completed. Once the phase transition occurs, cells must be kept from regressing. Therefore, although transitions are triggered by transient signals, they are irreversible processes and cells do not revert to an earlier state after the signal disappears. The irreversibility of cell cycle transitions is commonly attributed to the thermodynamic irreversibility of protein degradation, as is the case for cyclins and other cell cycle factors [26]. However, theoretical models, supported by many experimental observations, argue that feedback signals from reaction networks must exist to account for the irreversibility of cell cycle transitions [27], [28], [29].

Regarding the question raised above, p19 has a unique feature that distinguishes it from the remaining INK4 and makes it a likely candidate for contributing to the directionality of the cell cycle. p19 protein levels accumulate periodically during the cell cycle under normal conditions, a feature reminiscent of cyclins [30]. The cyclin D/CDK4,6 complexes perform a dual role in cell cycle regulation, via their specific pRb-directed kinase activity, and as a reservoir of the Cip/Kip proteins [17]. Therefore, the periodic expression of p19 could significantly affect the extent of active cyclin D-CDK4/6 complexes, and thus help ensure the physiological length of G1 phase.

It has been reported that the ubiquitin-dependent proteasome-mediated degradation of p19 might determine its periodic expression during the cell cycle [31]. However, both p19 mRNA and protein levels peak at the G1/S transition and decline again at the beginning of the G2 phase, indicating the existence of additional, transcriptional mechanisms of p19 regulation [30], [32], [33]. Although p18 mRNA was also reported to be induced by mitogens, only the kinetics of the p19 protein accumulation seemed to correlate tightly with its mRNA expression.

Here, evidence is presented that E2F1 can induce the expression of p19 through activation of the p19 promoter. Two E2F1-binding elements were located and characterized within the regions −635/−628 and −685/−678 from the translation initiation site (TIS). Transactivation of the p19 promoter by E2F1 was maximal only when both sites were present. Ablation of this regulation reduced p19 levels and restricted its expression during the cell cycle, reflecting the contribution of a transcriptional effect of E2F1 on p19 periodicity. Conversely, nuclear accumulation of E2F1 upregulated p19 and extended its temporal expression during most of the cycle. Finally, specific blockage of E2F1 binding to the p19 promoter caused a significant increase in cell proliferation and an altered cell cycle phase distribution.

E2F1-mediated regulation of p19 expression thus constitutes a negative feedback mechanism that limits CDK4,6 activity in late G1/S contributing to the proper coordination of the cell cycle and could represent and additional mechanism to control the proliferative-promoting function of E2F1.

## Results

### E2F1, E2F2 and E2F3 induce p19 gene expression at the transcriptional level

**Figure 1:**
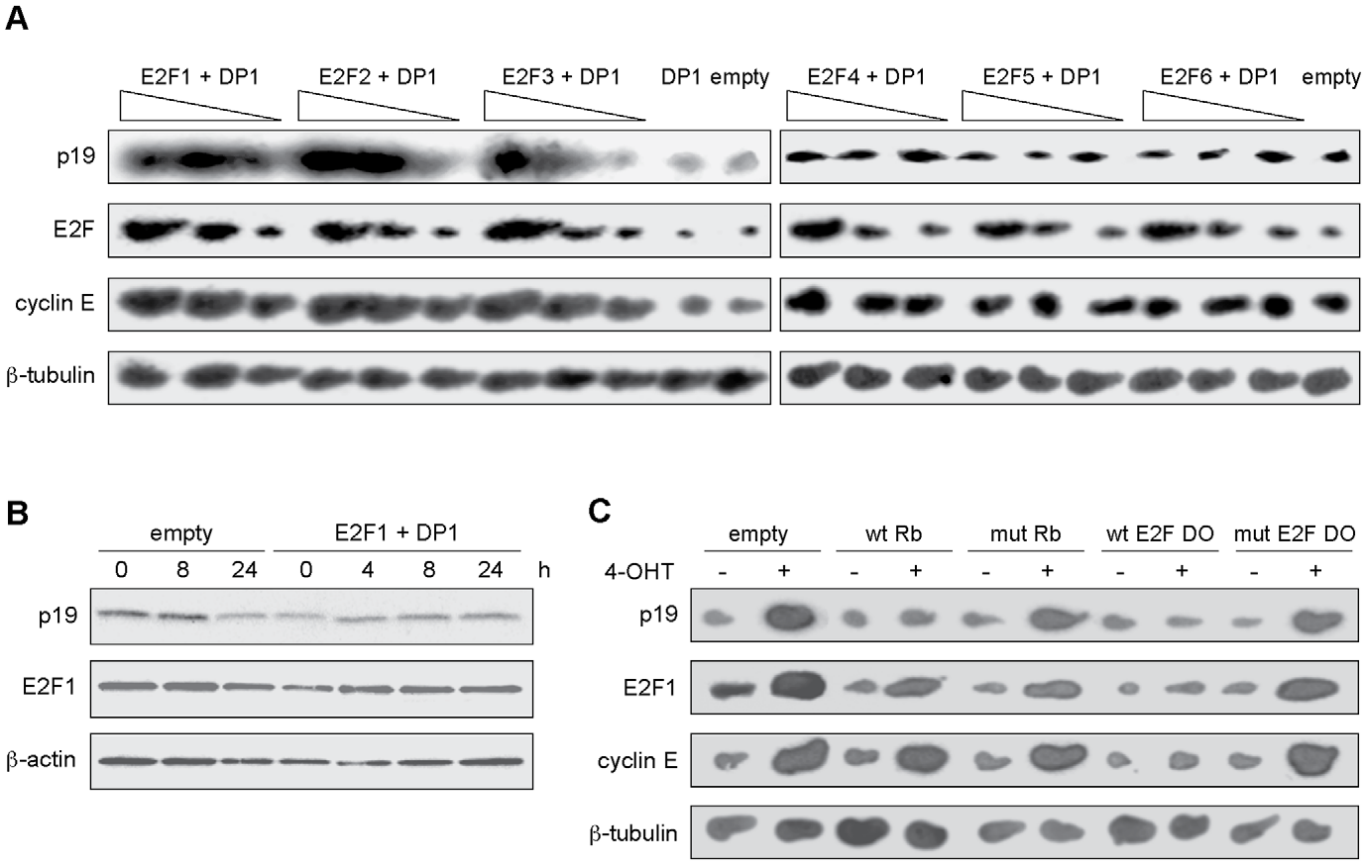
p19INK4d is induced by E2F1, 2 and 3. **A**. BHK-21 cells were cotransfected with pCMV, E2Fs and DP1 expression vectors (0.5–2.5 µg) and pBabe-Puro (0.5 µg), with total DNA amounts normalized with pCMV. Total RNA from puromycin-resistant cells was extracted and subjected to northern blot analysis. **B**. HEK-293 cells were cotransfected with E2F1 and DP1 expression vectors (2 µg) and cell lysates (100 µg) were immunoblotted. **C**. BHK-ER-E2F1 cells were transfected as indicated with pCMV, wild-type or mutant pRB expression vectors (2.5 µg) or wild-type or mutant E2F DO (100 nM), and pBabe-Puro (0.5 µg). After 24 h, cells were treated with 4-OHT for another 24 h. Total RNA was extracted from puromycin-resistant cells and subjected to northern blot analysis. Results showed in Figures are representative of at least two independent experiments.

The periodic expression of p19 during the cell cycle suggested that cell cycle-modulated transcription factors such as E2F family members could be involved in its regulation. To test this hypothesis, p19 expression levels were examined in BHK-21 fibroblasts transiently transfected with expression vectors encoding the E2F1 to E2F6 proteins and the E2F coactivator DP1 to ensure maximal E2F activity. Northern blot analysis showed that overexpression of E2F1, E2F2 or E2F3 upregulated p19 mRNA levels, whereas E2F4, E2F5 or E2F6 did not (Figure 1A). Likewise, cyclin E, a well-known E2F target, was only induced after E2F1, E2F2 or E2F3 transfection. These results are consistent with the potent transactivation ability of this E2F subclass [5]. Since E2F1 displayed the strongest stimulatory effect on p19 expression, subsequent experiments focused on this E2F family member. E2F1/DP1 overexpression caused a reproducible increase in p19 protein levels in HEK-293 cells (Figure 1B). The E2F1-mediated induction of p19 mRNA and protein was also observed in human cell lines, namely WI-38 fibroblasts, SH-SY5Y neuroblastoma and 293 embryonic kidney cells (data not shown).

To further study the regulation of p19 by E2F1, stable clones of BHK-21 cells were established which constitutively express an estrogen receptor-E2F1 fusion protein (BHK-ER-E2F1). The ER-E2F1 fusion protein is normally excluded from the nucleus and therefore transcriptionally inactive. Upon tamoxifen (4-OHT) addition, the protein rapidly enters the nucleus and induces E2F1 target genes [34], [35]. E2F nuclear accumulation by 4-OHT treatment resulted in a significant increase in p19 mRNA levels (Figure 1C). In cells with activated E2F1 expression, the mRNA levels of two E2F1 target genes, cyclin E and E2F1 itself, were also upregulated (Figure 1C). Circular dumbbell decoy oligonucleotides (DO) containing the E2F consensus sequence were used to sequester cellular E2F-1 away from its target gene promoters [36]. p19 upregulation was significantly impaired in BHK-ER-E2F1 cells transfected with wild-type E2F DO but was unaffected in those transfected with mutant E2F DO. Moreover, overexpression of wild-type tumor suppressor pRb, but not a mutant defective in E2F binding, partially blocked p19 mRNA induction as expected due to the ability of pRb to inactivate E2F1 (Figure 1C). Thus, E2F1 overexpression or nuclear accumulation stimulated p19 transcription, an effect that was prevented by two different strategies that inhibit E2F activity.

**Figure 2:**
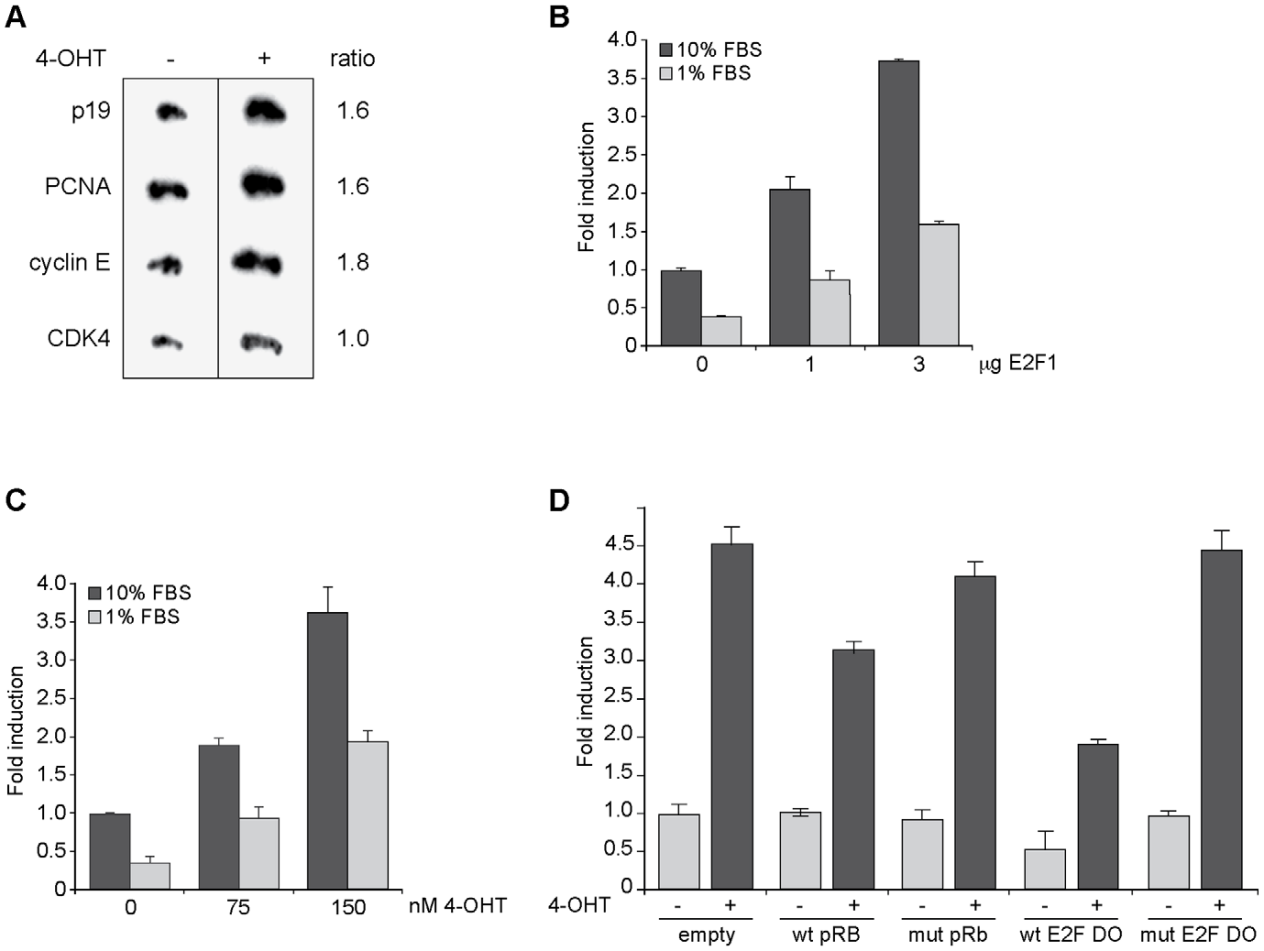
E2F1 increases the transcriptional activity of p19INK4d. **A**. BHK-ER-E2F1 cells were treated with 4-OHT for 8 h and subjected to nuclear run-on assay. Transcription rates of the indicated genes were normalized to that of β-tubulin. Results are representative of two independent experiments. **B–D**. Indicated cells were cotransfected with p19CAT (4.4 µg) and pCEFL-β-galactosidase (5 µg) reporter plasmids. CAT activity was determined 48 h after transfection and normalized to β-galactosidase activity. BHK-21 cells were transfected with reporter plasmids and 1 or 3 µg of E2F1 expression vector and grown in medium containing 10% or 1% FBS (**B**). BHK-ER-E2F1 cells were transfected with reporter plasmids, grown in medium containing 10% or 1% FBS, and treated with 4-OHT as indicated (**C**). BHK-ER-E2F1 were transfected with wild-type or mutant pRB expression vectors (6 µg) or wild-type or mutant E2F DO (100 nM) for 24 h. Cells were transfected with reporter plasmids for another 24 h before 4-OHT treatment (**D**). In panels **B**, **C** and **D** values are the average ± SD of three independent experiments, each performed in triplicates.

To determine whether E2F1 regulates p19 expression at the transcriptional level, run-on assays were performed. p19 transcription showed a 1.6-fold increase in BHK-ER-E2F1 cells treated with 4-OHT for 8 h compared to untreated cells. As expected, the transcription of E2F targets cyclin E and PCNA was comparably stimulated (Figure 2A).

A reporter was constructed containing 2307 bp derived from the genomic region upstream of the p19 TIS and the resulting plasmid p19CAT was transiently co-transfected into BHK-21 cells with increasing amounts of E2F1 expression vector. E2F1 caused a dose-dependent induction of p19 promoter activity (Figure 2B). Similarly, 4-OHT treatment of p19CAT-transfected BHK-ER-E2F1 cells resulted in a significant increase of CAT activity (Figure 2C). These experiments were also performed in cells growing in medium with 1% FBS to exclude any potential effects from serum factors. Although higher reporter activities were observed in cells grown in 10% FBS, E2F1 stimulated p19 transactivation in both serum conditions to a similar extent (Figs. 2B and 2C). These results suggest that E2F1 alone is sufficient to induce transcriptional activity of p19 promoter without other serum requirements.

Consistent with the results obtained for p19 mRNA levels, transfection with either E2F DO or wild-type pRb expression vector, but not their mutated versions, prevented p19 promoter activation by E2F in 4-OHT-treated cells (Figure 2D). Taken together, these results suggest that E2F1 regulates p19 gene expression.

### p19 promoter contains two functional E2F-binding sites

**Figure 3:**
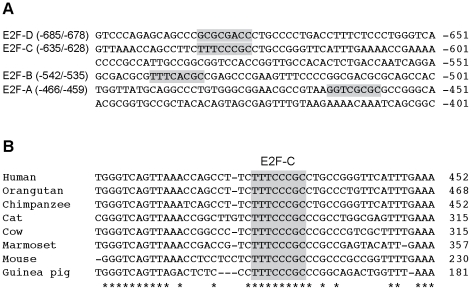
An E2F responsive element from the human p19 promoter is conserved across a wide range of mammalian species. **A**. Sequence of the human p19 promoter (−961/−1 from p19 translation initiation site) with putative E2F binding sites indicated by boxes. Deviations from the E2F consensus are shown by underlined letters. **B**. Sequence alignment of p19 promoter regions from different mammalian species using the ClustalW software. Conserved residues are marked by asterisks and the box indicates the E2F-C site.

Sequence analysis of the human p19 promoter revealed four putative E2F1 binding sites, referred to as E2F-A to E2F-D. These elements are located within the region −400/−700 bp from TIS and show various degrees of homology with the E2F consensus sequence (5′-TTT(C/G)(C/G)CGC-3′) (Figure 3A). Interestingly, the E2F-C site is not only a perfect match of the E2F consensus sequence, but genomic sequence analysis revealed that it is also perfectly conserved across a wide range of mammalian species (Figure 3B). This suggests that this element is likely to play an important role in the regulation of p19 expression.

**Figure 4:**
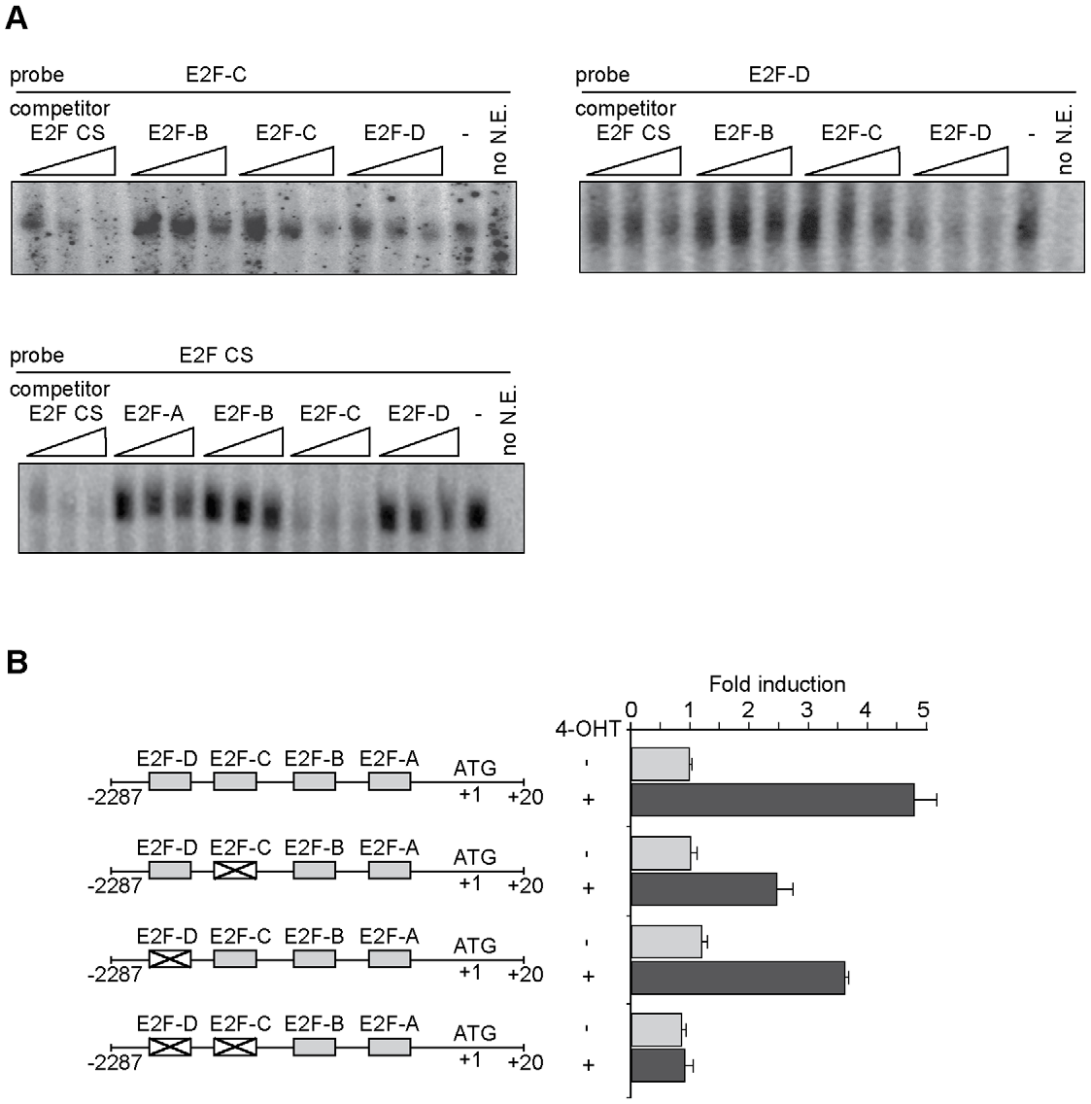
The p19INK4d promoter contains functional E2F binding sites. **A**. EMSA was performed using oligonucleotides corresponding to the E2F-C (*top panel*) or E2F-D (*middle panel*) sites or the E2F consensus sequence (E2F CS) (*bottom panel*) as radiolabeled probes. BHK-21 nuclear extracts (N.E.) were incubated with probes alone or in the presence of 50-, 500-, or 1000-fold molar excess of each of the indicated unlabeled competitors. Results are representative of at least two independent experiments. **B**. BHK-ER-E2F1 cells were cotransfected with 4.4 µg of the indicated CAT reporters and 5 µg of pCEFL-β-galactosidase for 24 h. Cells were treated with 4-OHT as indicated. CAT activity was determined 48 h after transfection and normalized to β-galactosidase activity. Values are the average ± SD of two independent experiments, each performed in quadruplicate.

To address the functionality of these sites EMSA was performed using radiolabeled probes containing the sequences from the putative regulatory elements. Probes E2F-C and -D were able to form a protein-DNA complex similar to that seen using an E2F consensus sequence (E2F CS) (Figure 4A). Moreover, unlabeled oligonucleotides containing either E2F-C or -D, as well as E2F CS, competed for binding to E2F proteins. In contrast, E2F-A and -B probes failed to form any specific complexes with E2F and to efficiently compete for binding (Figure 4A and data not shown). These results suggest that E2F-C and E2F-D are E2F binding sites, with the former having the highest affinity for the transcription factor.

To examine the role of the E2F-C and -D sites on p19 promoter activity, p19CAT-derived reporters were constructed in which the E2F-C and -D sequences were mutated alone or in combination. Mutation of either element caused a reduction in p19 promoter activation by E2F1 in BHK-ER-E2F1 cells treated with 4-OHT, as compared to the wild-type reporter. Consistent with its highest affinity, the contribution of the E2F-C site to the overall transactivation of p19 by E2F was greater than that of the E2F-D element (Figure 4B). Finally, the combined mutation of both sites completely abolished the p19 promoter responsiveness to E2F1. Thus, E2F-C and E2F-D are bona fide E2F response elements that appear to be sufficient to account for the transcriptional upregulation of the p19 promoter by E2F1.

### p19 periodic expression in the cell cycle is dependent on E2F

**Figure 5:**
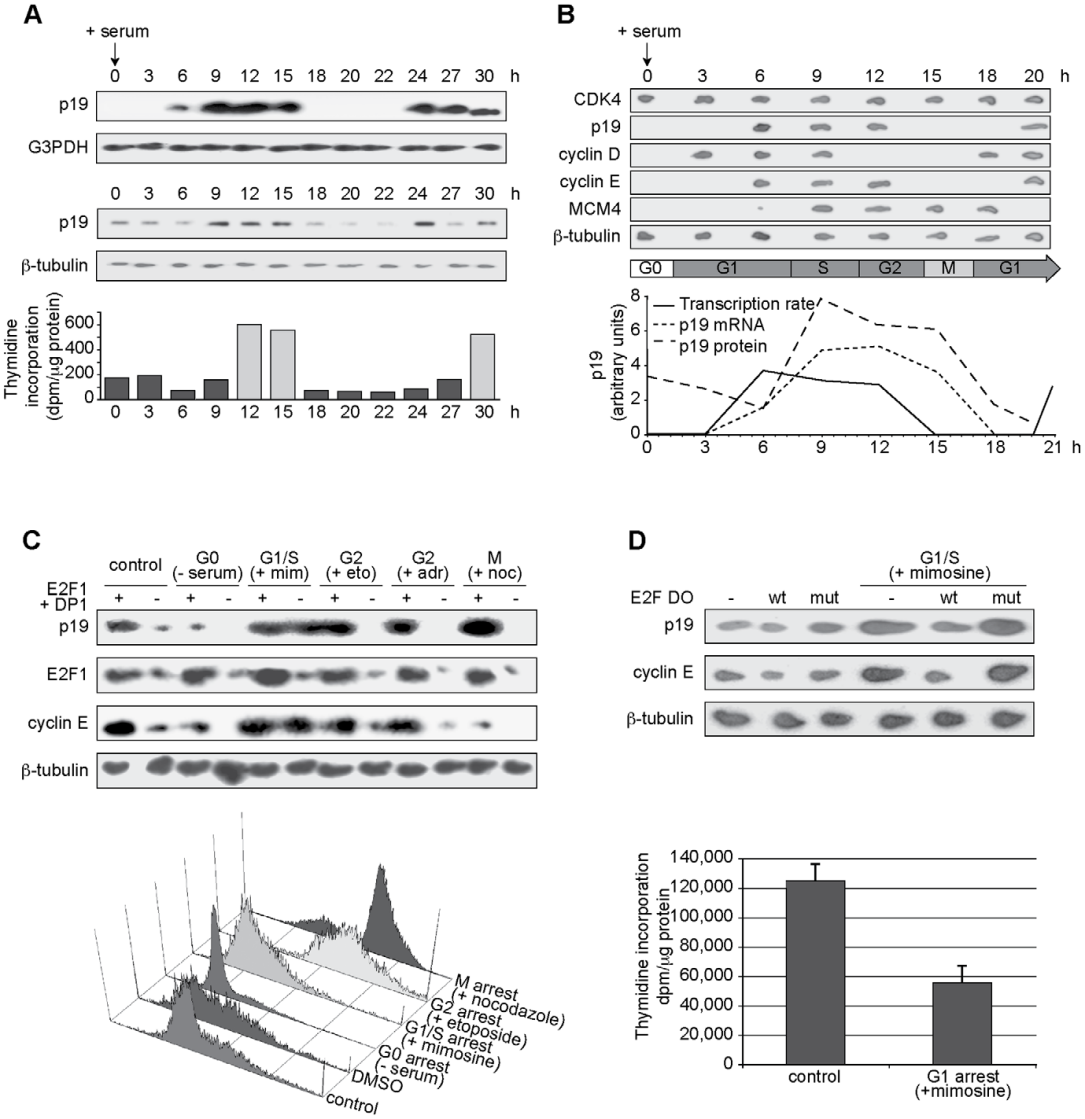
p19 periodic expression during cell cycle is dependent on E2F1. **A.** BHK-21 cells were synchronized by serum deprivation. Total RNA was extracted at the indicated times following serum restoration and subjected to northern blot analysis (*top panel*). Corresponding dishes were subjected to immunoblotting (*middle panel*) and thymidine incorporation analysis (*bottom panel*). **B**. Synchronized BHK-21 cells were subjected to nuclear run-on assay at the indicated times after cell cycle re-entry (*top panel*). Levels of p19 protein, mRNA and *de novo* mRNA synthesis are shown (*bottom panel*). **C**. BHK-21 cells were treated to induce cell cycle arrest in different phases (for G0 arrest: 1% serum, for G1/S: 200 µM mimosine (mim), for G2: 2.5 µM etoposide (eto) or 100 µM adriamycin (adr), for M: 0.1 µg/ml nocodazole (noc)) and cotransfected with expression vectors for E2F1 (2.75 µg), DP1 (2.5 µg) and pBabe-Puro (0.5 µg). Total RNA from puromycin-resistant cells was extracted and subjected to northern blot analysis (*top panel*). Corresponding dishes were subjected to flow cytometry (*bottom panel*). **D**. BHK-21 cells were transfected with 100 nM wild-type or mutant E2F DO as indicated for 18 h. Cells were then arrested in G1 phase by treatment with 200 µM mimosine for 36 h. Total RNA was extracted and subjected to northern blot analysis (*top panel*). Corresponding dishes were subjected to thymidine incorporation assay (*bottom panel*). Values are the average ± SD of three independent experiments. Results showed in panels **A**, **B**, **C**, and **D** are representative of at least two independent experiments.

As previously reported for other cell types, p19 mRNA and protein levels oscillate throughout the cell cycle in proliferating normal diploid BHK-21 fibroblasts, with the highest levels observed in late G1 and S phases (Figure 5A) [37]. The tight correlation between its mRNA and protein levels suggests that p19INK4d is a short-lived protein. The periodic oscillation of the p19 protein during the cell cycle was indeed attributed to its short half-life (2-2.5 h), caused by its ubiquitin-dependent proteasome-mediated degradation [31], [33]. Although the posttranslational regulation of p19 has been studied in detail, the contribution of a pretranslational mechanism in its periodic expression remains to be explored. The cyclic variation of p19 mRNA levels indicates that its synthesis or stability must be regulated in a cell cycle-dependent manner.

To determine whether p19 mRNA periodicity is regulated at the transcriptional level, the rate of transcription initiation of p19 and several genes involved in the G1/S transition was determined by run-on assays in synchronized BHK-21 cells (Figure 5B). Cell cycle progression was monitored by analysis of thymidine incorporation (Figure 5A, bottom) and expression levels of known cell cycle markers (data not shown). p19 transcription was undetectable in starved, quiescent cells and cells entering G1 phase, then increased rapidly in late G1/S phases to finally decline again as cells entered the following cycle. The relative abundance of p19 mRNA determined by northern blot closely followed this time-dependent pattern of transcription. These results argue that the oscillatory behavior of p19 during the cell cycle is mostly due to regulation of p19 transcription and not mRNA stability.

The cyclin D-dependent kinase CDK4 was detected in serum-starved cells and throughout the ensuing cell cycle. By contrast, cyclin D1 was barely detected in G0 cells but actively transcribed during G1 phase in response to mitogen stimulation, consistent with progressive cyclin D-dependent assembly and allosteric activation of cyclin D1/CDK holoenzymes as cells approach the G1/S boundary. Interestingly, the kinetics of p19 transcription were similar to that of cyclin E. Cyclin E is an early E2F1-target gene whose expression is essential to allow induction of several E2F1-responsive genes that are required to drive cells through the G1/S transition and initiate DNA replication.

The fact that E2F1 induced p19 expression and that p19 and cyclin E showed similar transcription kinetics suggested that the periodic expression of p19 during the cell cycle could be a consequence of the increased E2F1 activity during late G1/S phases. To examine this possibility, BHK-21 cells transfected with E2F1 and DP1 expression vectors were arrested, by treatment with specific inhibitors, at each phase of the cell cycle, as determined by flow cytometric analysis (Figure 5C). Ectopic expression of E2F1 induced p19 levels in all cell cycle phases, including those in which its expression is usually undetectable (Figure 5C). The correlation between p19 transcription and free E2F1 levels, regardless of cell cycle position, supports a role for E2F1 as the major regulator of the oscillatory behavior of p19 throughout the cell cycle, independently of other cell cycle-associated factors. However, the contribution of a factor like E2F4 or E2F5 that, through the same E2F1-binding sites, might repress p19 in situations of low or absent E2F1 cannot be excluded. Finally, a wild-type E2F DO but not a mutated version blocked p19 and cyclin E mRNA upregulation in BHK-21 cells arrested by mimosine treatment at the G1/S boundary, when E2F1 activity is elevated. The cell cycle arrest by mimosine was assessed by thymidine incorporation (Figure 5D). These results support a model in which p19 expression in G1/S is regulated by E2F1 transcriptional activity and this mechanism is responsible for p19 periodicity during the cell cycle.

### E2F1 sequentially induces cyclin E and p19 during the cell cycle

**Figure 6:**
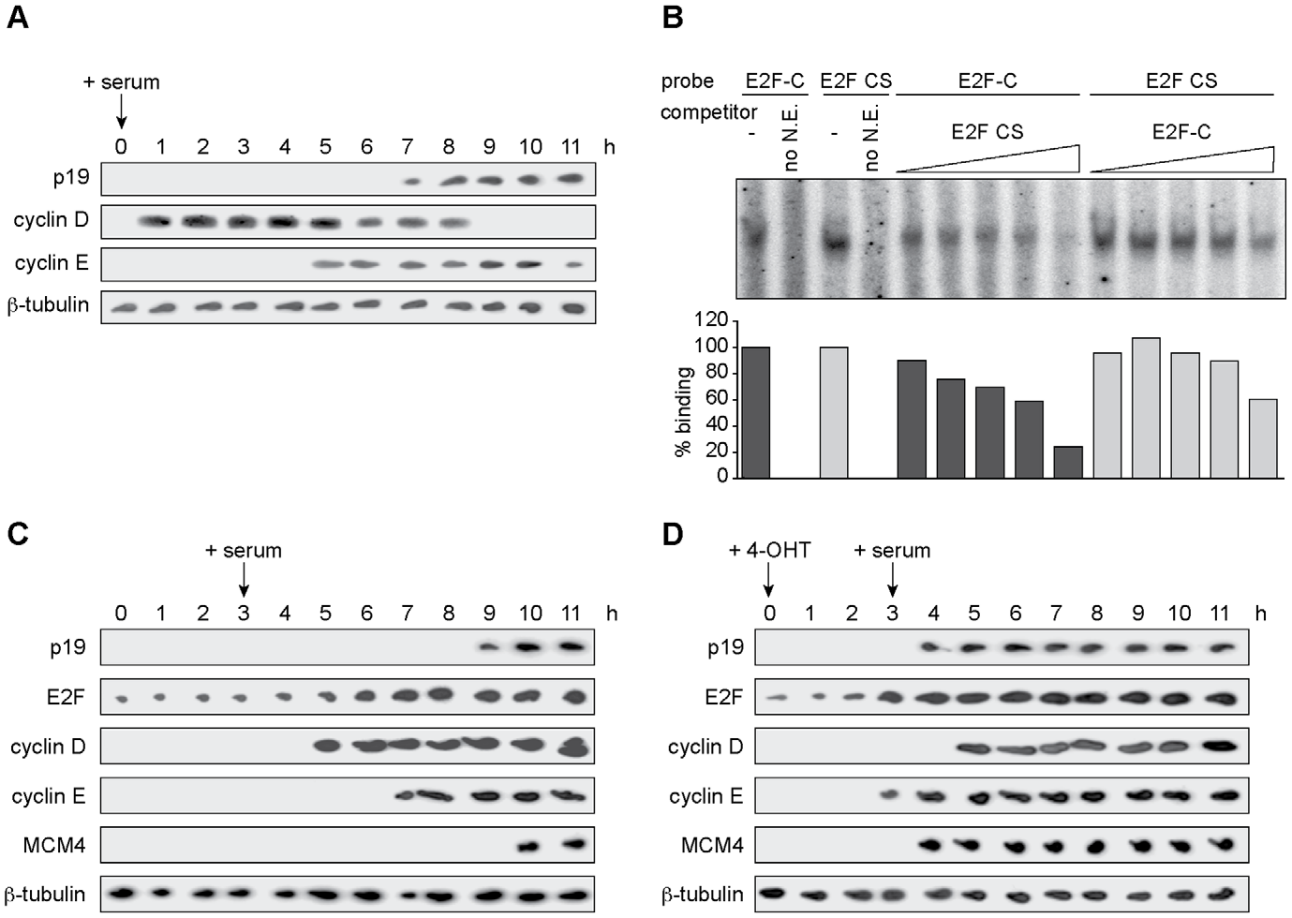
E2F1 sequentially induces cyclin E and p19 during the cell cycle. **A**. WI-38 cells were synchronized by serum deprivation. Total RNA was extracted at the indicated times following serum restoration and subjected to northern blot analysis. **B**. EMSA was performed using oligonucleotides corresponding to the E2F-C site or the E2F consensus sequence (CS) as radiolabeled probes. WI-38 nuclear extracts (N.E.) were incubated with probes alone or in the presence of 20-, 50-, 100-, 200-, or 500-fold molar excess of the indicated unlabeled competitors. Relative quantification of DNA-protein complexes is shown (*bottom panel*). **C** and **D**. Synchronized BHK-ER-E2F1 cells were untreated (**C**) or treated with 4-OHT (**D**) for 3 h before cells were stimulated to re-enter the cycle. Total RNA was extracted at indicated time points and subjected to northern blot analysis. Results are representative of at least two independent experiments.

The kinetics of cyclin E and p19 mRNA expression during the cell cycle were further explored using WI-38 cells arrested in early G1 phase by serum deprivation for 24 h. When cells re-entered the cell cycle synchronously by restimulation with serum, transcription of cyclin E started at least two hours before p19 transcription was initiated (Figure 6A). Thus, during cell cycle progression there is a temporal separation of the expression of these two antagonistic genes.

A possible mechanism to explain the sequential transactivation of cyclin E and p19 by the same transcription factor E2F1 would be that the cyclin E and p19 E2F binding sites have different affinities for the E2F1 protein. As its physiological levels progressively increase in response to mitogenic signals, the E2F1 protein would bind first to the cyclin E promoter and then to elements in the p19 regulatory region. To determine the relative affinities for E2F1 of the E2F sites from the cyclin E and p19 promoters EMSA were performed. Supporting this hypothesis, competition experiments using unlabeled oligonucleotides containing the E2F binding sites from the cyclin E or the p19 promoters showed that the former has greater affinity for the E2F1 protein (Figure 6B). In this situation, E2F1 overexpression should saturate the E2F response elements in both gene promoters and reduce the temporal separation in their expression. BHK-ER-E2F1 cells were then arrested by serum deprivation and nuclear translocation of ectopic E2F1 was induced by 4-OHT treatment for 3 hours before cells were allowed to re-enter the cell cycle. Cyclin E and p19, as well as Mcm4, another E2F target gene, were induced earlier by overexpressed E2F1 and with similar kinetics, compared to cells with only endogenous E2F1 expression (Figure 6C) [38]. In contrast, the expression of cyclin D1, an E2F-unresponsive gene, remained unaffected by this treatment.

Collectively, these results suggest that E2F1 binds with higher affinity to elements in the cyclin E promoter than to those in the p19 promoter. This observation offers a potential mechanism to explain temporal differences in the expression of both genes during cell cycle progression. However, other possibilities cannot be excluded such as the existence of multiple E2F binding sites or the proximity of these sites to the transcription start point of the cyclin E gene [39].

### Periodic expression of p19 contributes to proper cell cycle regulation

The results described above demonstrate that E2F1 regulates p19 gene transcription and that the periodic expression of p19 is a direct consequence of this regulation. Moreover, E2F1-mediated induction of p19 occurs later than that of cyclin E, a canonical proliferative event.

**Figure 7:**
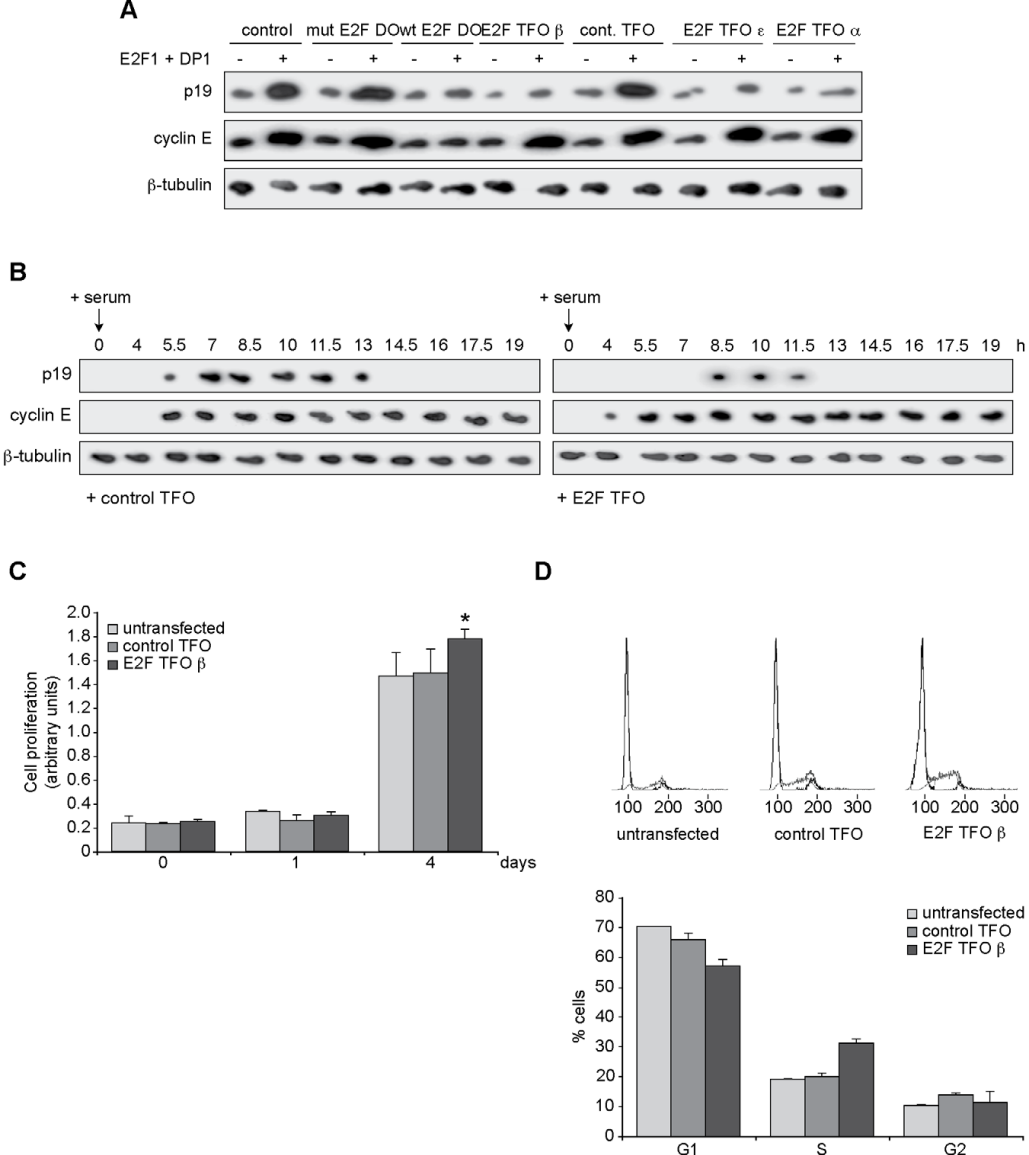
Periodic expression of p19 contributes to proper cell cycle regulation. **A**. HEK-293 cells were transfected with E2F1 and DP1 expression plasmids and the indicated oligonucleotides (100 nM for DO and 500 nM for TFO) for 24 h. Total RNA was extracted and subjected to northern blot analysis. **B**. WI-38 cells were transfected with control or E2F TFO β and synchronized by serum deprivation 24 h after transfection. Total RNA was extracted at the indicated times following serum restoration and subjected to northern blot analysis. **C**. WI-38 cells were transfected with the indicated TFO for the indicated days and thymidine incorporation was assayed at the indicated time points. Data was compared using Mann-Whitney test (SPSS 11.5.1 LEADTOOLS). Asterisk indicates p<0.05. **D**. Corresponding dishes from day 4 were subjected to flow cytometry (*top panel*). Results are quantitated in bar graph (*bottom panel*). In **A**, **B**, **C** and **D** *panels r*esults are representative of at least three independent experiments.

To gain insight into the physiological consequences of the E2F1 induction of p19, its binding to the p19 promoter was prevented using triple-helix forming oligonucleotides (TFO). TFO were designed to bind to purine/pyrimidine rich-sequences near the E2F-C and -D sites and specifically interfere with E2F1 regulation of p19 expression, affecting neither E2F1 intracellular levels nor its transcriptional activity on other target gene promoters. To analyze the efficiency and specificity of these TFO, HEK-293 cells were transfected with an E2F1 expression vector together with one of several TFO or an E2F DO for 24 h. Three different E2F TFO (α, β, or ε) but not a scrambled version reduced p19 induction in response to E2F1 overexpression, whereas none of them affected cyclin E or β-tubulin mRNA levels (Figure 7A). Only cells transfected with E2F DO exhibited an impaired induction of both p19 and cyclin E by E2F1. Similar results were observed at 96 h after transfection (data not shown). A time course analysis of p19 expression in normal diploid human WI-38 fibroblasts transfected with TFO showed that p19 mRNA expression was both significantly reduced and delayed by E2F TFO β (Figure 7B). TFO β however did not significantly affect cyclin E expression levels or timing.

E2F TFOs were then used to address the physiological relevance of p19 regulation by E2F1 during the cell cycle. Since E2F1 expression has been shown to stimulate cell proliferation, p19 upregulation might therefore function to limit the cell cycle promoting effect of E2F1. To test this hypothesis, WI-38 cells were transfected with TFO and cell proliferation was determined by MTT assay. Cells in which E2F1-mediated upregulation of p19 was prevented by TFO showed a 20% increase in MTT activity 4 days after transfection, as compared to untransfected cells or cells transfected with control oligonucleotides (Figure 7C). Flow cytometric analysis showed an increase in the S phase population and a concomitant reduction in the proportion of cells in G1 phase in E2F TFO transfected cells. This observation indicates that when E2F1 fails to induce p19, there is a reduction in the relative duration of the G1 phase and/or an increase in that of the S phase (Figure 7D and 6E). Taken together, these results strongly suggest that p19 plays a role in limiting the cell cycle promoting activity of E2F1, providing a fine-tuning mechanism for the kinetics of the eukaryotic cell cycle.

## Discussion

Although cell fate is highly influenced by environmental signals during the early G1 phase, past the restriction point, when cells commit to a new round of division, they will progress through the cell cycle forwardly until mitosis is completed. Thus, cell cycle progression requires an appropriate balance of positive and negative regulatory factors, such as cyclins and CDKs, and CKIs, respectively. These cell cycle regulators must ensure the completion of each phase as well as the irreversibility of each transition. Their expression must then fluctuate in a coordinated manner, in accordance with each division phase.

As mentioned earlier, the fact that p19 is the only member of the INK4 family whose mRNA and protein levels fluctuate periodically during the cell cycle suggested that it might play a role in cell cycle regulation in normal dividing cells [32]. This prompted the study on the impact that this pattern of expression might play in the cell cycle, as well as on the underlying transcriptional mechanisms that lead to it. The short half-life of the p19 protein and the fact that, because of this, its levels closely reflect those of its mRNA emphasized the importance of understanding the regulation of the p19 gene at the transcriptional stage.

Several transcription factors had previously been reported to regulate p19 gene expression. p19 was induced by FOXO during G1 arrest caused by Akt inactivation, by Stat3 in macrophage proliferation inhibition by IL-10, by Sp1 in multiple cell lines treated with HDAC inhibitors and by AML-1 en megakaryocytes [40], [41], [42], [43]. Repression of p19 by Egr1 was reported in prostate cancer cells [44]. However, none of these transcription factors appear to be able to account for the periodic expression of p19.

The studies presented here show that E2F1 induces the p19 gene. E2F1 overexpression upregulated p19 in normal cycling cells as well as in cells arrested at all cell cycle phases. The latter result indicates that it is a transcriptional effect of the increased levels of free E2F1 and not an indirect consequence of E2F1 overexpression on cell cycle position. These results are consistent with a previous report showing that both E2F1 and E2F2 were capable of upregulating p19 mRNA levels, among other cell cycle regulators [45]. Another report identified an E2F binding site in the p19 gene promoter but the authors failed to detect a significant upregulation of p19 by E2F1 using a reporter construct containing the −3814/−2 region from the TIS from the p19 promoter [46]. The reasons for this discrepancy remain to be explored.

The regulation of p19 expression by E2F1 could be the mechanism responsible for its periodicity. Several evidences support this conclusion. E2F1 transcriptional activity and p19 induction displayed similar kinetics. Moreover, E2F1 forced expression in cycling cells altered p19 expression kinetics, leading to an early induction during the cell cycle. Finally, when binding of endogenous E2F1 to the p19 promoter was prevented using TFO, p19 levels and duration of expression were significantly reduced.

E2F1 has a well-characterized proliferative function through the induction of cyclin E and other genes whose products are involved in the G1 and S phases. In contrast, p19 has been described as an antiproliferative factor due to its ability to inhibit the cyclin D/CDK4,6 complexes. Thus, the data discussed here show a dual effect of E2F1, which can lead to both positive and negative control of cell cycle progression. This apparent conundrum could be explained by different timing of expression of proliferative and antiproliferative genes. Kinetic analysis of the E2F target genes showed a 2-hour delay between the expression of p19 and that of cyclin E, supporting this hypothesis.

Finally, the physiological relevance of this mechanism was explored using TFO that specifically block the binding of E2F to its response elements in the p19 promoter, without affecting other E2F target genes. Cell proliferation and cell cycle distribution analysis showed that the E2F-p19 pathway regulates temporal variables of the cell cycle. More specifically, inhibition of this pathway stimulated cell proliferation and increased the relative fraction of cells in S phase.

**Figure 8:**
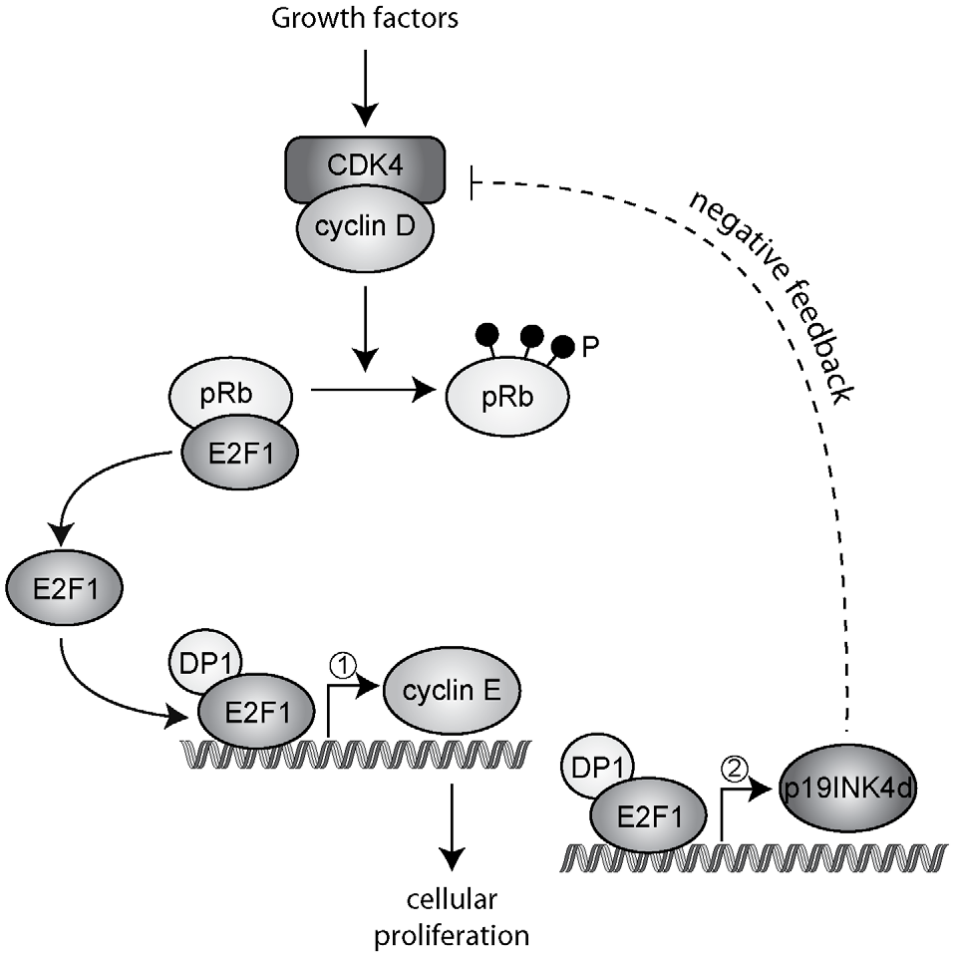
p19 mediates a negative feedback mechanism that regulates E2F activity. A simplified model of normal cell cycle progression. Following phosphorylation of pRb, free E2F induces p19 through binding to its promoter. This induction is delayed, compared to that of cyclin E (indicated by numbers 1 and 2), due to differences in the affinity of the E2F binding sites in both promoters. The E2F-dependent accumulation of p19 inhibits the CDK4 containing complexes, bringing the G1 phase to an end.

Taken together, the results described here support a model of normal cell cycle progression in which, following phosphorylation of pRb, free E2F induces cyclin E, among other target genes. Once cyclinE/CDK2 takes over as the cell cycle driving kinase activity, the induction of p19 mediated by E2F1 leads to inhibition of the CDK4,6-containing complexes, bringing the G1 phase to an end (Figure 8). Thus, this is the first description of a regulation of p19 by a transcription factor, E2F, which can explain its cyclic pattern of expression. Moreover, this pattern appears to be involved in a new feedback mechanism mediated by p19 that regulates E2F activity, the duration of the G1 phase and entry into S phase. This regulation contributes to terminating the proliferative activity of the G1 cyclin/CDK complexes and provides an additional mechanism to limit E2F activity. However, its involvement in the mechanisms that guarantee the irreversibility of the G1/S transition remains to be established.

E2F target genes encoding antiproliferative functions have previously been identified. p27Kip1 was similarly shown to be induced by E2F1 and regulate cell cycle progression in normal, physiological conditions [47]. In other reports, both p21Cip1 and p27Kip1 were shown to be regulated by elevated E2F activity and control cell cycle progression [48], [49].

Thus, the induction of p19 by E2F1 is a normal event that takes place during cell cycle progression. This step could be part of a regulatory network that contributes to the fine-tuning of the cell cycle.

p18 mRNA levels have also been reported to fluctuate during the cell cycle and to be induced by E2F [45], [50]. However, p18 protein levels do not reflect its mRNA levels, suggesting that this pattern of expression does not have a biological significance in cell cycle regulation. It could be speculated that the ability of the p18 and p19 promoters to respond to E2F was acquired early during evolution and that this regulation might confer an advantage in a different cellular context. Since p19 is the only INK4 family member with the shortest half-life, the most parsimonious evolutionary scenario would indicate that this characteristic was acquired later during evolution. It is the combination of both attributes, short half-life and E2F responsiveness, which confers p19 protein levels a fluctuating periodic behavior that is important in cell cycle control.

## Materials and Methods

### Cell lines, drug treatments and transfections

BHK-21 and HEK-293 cell lines were grown in DMEM (Invitrogen) supplemented with 10% fetal bovine serum (FBS), 100 U/ml penicillin, 100 µg/ml streptomycin, 100 mM non-essential amino acids, and 2 mM glutamine at 37°C in a 5% CO_2_ humidified atmosphere. WI-38 cells were maintained in MEM (Invitrogen) supplemented as indicated above. When indicated, cells were synchronized by serum deprivation (growth in medium containing 1% FBS) for 24–36 h, then allowed to re-enter the cell cycle by addition of regular growth medium. Stable BHK-ER-E2F1 cells were previously described [35]. E2F1 activity was induced in these cells by treatment with 300 nM 4-OHT (Sigma) for 5 h. When indicated, cells were incubated in medium with 0.5% FBS or treated with 200 µM mimosine, 2.5 µM etoposide, 100 µM adriamycin, 0.1 µg/ml nocodazole (Sigma).

Cells were transfected using Lipofectamine 2000 (Invitrogen) according to the manufacturer's instructions. Decoy and triplex forming oligonucleotides (Bio-Synthesis) were transfected at a final concentration of 100 and 500 nM, respectively. When indicated, expression plasmids were cotransfected with pBabe-Puro and cells were selected with 1.5 µg/ml puromycin (Sigma) for 60 h before assayed.

### Oligonucleotides

Circular dumbbell double-stranded decoy oligonucleotides: wild-type E2F decoy: 5′-ATGCGCGAAACGCGTTTTCGCGTTTCGCGCATAGTTTTCT-3′ (where the underlined sequences correspond to the E2F consensus biding site) and mutant E2F decoy: 5′-ATAATCTAAACGCGTTTTCGCGTTTAGATTATAGTTTTCT-3′ oligonucleotides were annealed and ligated for 24 h at 16°C with 1 unit of T4 DNA ligase (Invitrogen). The patent application for triplex-forming oligonucleotides sequences is pending. The oligonucleotides were from Bio-Synthesis, Inc.

### RNA extraction and northern blot analysis

RNA extraction and northern blot analysis were previously described [51]. Briefly, 10–20 µg of total RNA were denatured, electrophoresed in 1% glyoxal-agarose gels, and transferred to nylon membranes (Hybond N, Amersham). Membranes were sequentially hybridized with the indicated [^32^P]-labeled probes and radioactivity was detected using a PhosphorImager (FujiFilm BAS-1800II).

### Immunoprecipitation and western blot analysis

Cells were lysed with RIPA buffer (50 mM Tris-HCl pH 7.5, 150 mM NaCl, 1% Nonidet P-40, 0.5% sodium deoxycholate, 0.1% SDS, 100 µg/ml phenylmethylsulfonylfluoride, 60 µg/ml aprotinin and 1 mM sodium orthovanadate). Equal amounts of proteins (100 µg) were mixed with anti-human p19 monoclonal antibody (P0999-55A, USBiologicals) and protein A/G-agarose beads (Santa Cruz) for 1 h at 4°C. Beads were washed four times with PBS, resuspended in sample buffer containing 2% SDS and 30 mM β-mercaptoethanol, and boiled for 3 min. Proteins were resolved in 10% polyacrylamide gels and analyzed by immunoblotting using anti-p19 antibody. E2F1 (sc-251), actin (sc-47778), and secondary antibodies were from Santa Cruz. The signal was detected using enhanced chemiluminescence detection reagent (Amersham Life Sciences) and LAS-1000 Image Analyzer (FujiFilm).

### Nuclear run-on transcription assay

Nuclear run-on transcription assays were performed as previously described [52]. Nuclear RNA was radiolabeled, extracted and hybridized to nylon membranes (GeneScreen Plus, Perkin Elmer) previously slot-blotted with 10 pmoles of specific single-stranded DNA probes. Radioactivity was detected using a PhosphorImager (FujiFilm BAS-1800II).

### Reporter constructs and site-directed mutagenesis

A 2307 bp fragment of the human p19INK4d promoter (−2887/+20 from TIS) was amplified by PCR using the following primers containing XbaI and XhoI restriction sites (underlined): 5′-CGTCTAGATCTCCCCTGCTCTGTACCAC-3′/5′-TACTCGAGGAACCTCCTCCAGCAGCAT-3′. The fragment was cloned into the XbaI and XhoI sites of the pBLCAT6 reporter plasmid to create p19CAT. E2F sites in the human p19 promoter were mutated alone or in combination as follows: E2FC site: TTTCCCGC to TTTCCTAC (−630/−629 from TIS) and E2FD site: GCGCGACC to ATGCGACC (−685/−684). Site-directed mutagenesis was performed by Mutagenex, Inc.

### Reporter assay

Cells were transfected following the standard calcium phosphate precipitation method essentially as previously described [53]. Briefly, cells seeded in 6-well dishes were transfected with 4.4 µg CAT reporter plasmid, 5 µg of pCEFL-β-galactosidase and expression vectors when indicated. Total DNA amount was adjusted to 15.4 µg/well with non-specific DNA carrier. After 16 h, the medium was replaced by serum-free medium, and cells were further incubated for 24 h. Cells were then harvested and CAT and β-galactosidase activities were determined as previously described [54]. CAT activity was normalized to β-galactosidase activity.

### Electrophoretic mobility shift assay (EMSA)

EMSAs were performed as previously described [53]. Briefly, complementary single-stranded oligonucleotides were annealed and end-labeled with T4 polynucleotide kinase and 50 µCi of [γ-^32^P]-ATP (6000 Ci/mmol, 150 Ci/µl) at 37°C for 30 min. Nuclear extracts (5–20 µg), 150,000 dpm of labeled probe (0.07–0.1 pmoles) and 3 µg of poly[d(I–C)] were incubated in a total volume of 20 µl of TM buffer (50 mM Tris-HCl, pH 7.9, 12.5 mM MgCl2, 1 mM EDTA, 1 mM dithiothreitol, 20% glycerol) for 30 min at room temperature. When indicated, nuclear extracts were preincubated for 20 min at room temperature with excess of unlabeled competitor DNA before the addition of the labeled probe. Samples were loaded on native 5% polyacrylamide gels in 0.25X TBE. Gels were dried and radioactivity detected as described above. Double-stranded DNA probes and cold competitors used were: E2F-A (−473/−452 bp): 5′-GCCGTAAGGTCGCGCGCCGGGC-3′; E2F-B (−549/−528 bp): 5′-CGACGCGTTTCACGCCGAGCCC-3′; E2F-C (−642/−621 bp): 5′- AGCCTTCTTTCCCGCCTGCCGG-3′; E2F-D (−692/−671 bp): 5′-GCAGCCCGCGCGACCCTGCCCC-3′. The E2F consensus and mutant sequences used were: 5′-TCAGTTTTCGCGCCTAAACACAAAC-3′ and 5′-TCAGTTTTCGATCCTAAACACAAAC-3′, respectively.

### Thymidine incorporation, MTT assay and flow cytometry

Thymidine incorporation and MTT assay were previously described [32], [37]. Cells for flow cytometry were prepared as described [32]. DNA content and cell cycle distribution were analyzed using a FACScan flow cytometer (Becton Dickinson) and WinMDI 2.9 and Cylchred 1.0.2 software.
